# Characteristics and phylogenetic analysis of the complete chloroplast genome of *Rubus chingii* Hu 1925 from the family Rosaceae

**DOI:** 10.1080/23802359.2023.2268220

**Published:** 2023-11-20

**Authors:** Yuxian Li, Ying Qiu, Min Yang, Yongfei Yin, Min Li, Ying Zhang

**Affiliations:** a Traditional Chinese Medicine Department, Jilin Agricultural Science and Technology University, Jilin, PR China; b Department of Pharmacy, Anhui University of Chinese Medicine, Hefei, PR China

**Keywords:** *Rubus chingii*, complete chloroplast genome, phylogenetic analysis, Rosaceae

## Abstract

*Rubus chingii* Hu 1925 is an important medicinal vine shrub in the Rosaceae family, widely distributed in China and Japan. In this study, the complete chloroplast genome of *R. chingii* was sequenced and identified. The chloroplast genome was 155,563 bp in size with a total GC content of 37.06%. Two 25,749-bp inverted repeat (IRA and IRB) regions divided the genome as four sections, with the remainder forming a large single-copy (LSC, 85,322 bp) and a small single-copy (SSC, 18,743 bp) regions. This genome contained a total of 131 genes, of which 86 were protein-coding genes, 37 tRNA genes, and eight rRNA genes. The phylogenetic analysis showed that *R. chingii*, along with several other *R. longisepalus*, *R. tsangii*, *R. hirsutus*, *R. taiwanicola*, *R. rubroangustifolius*, and *R. glandulosopunctatus*, formed the monophylic group. Interestingly, the chloroplast genome structure we reported was different from the previously reported structure and provided richer phylogenetic analysis information in the *Rubus* genus compared to previous studies. The genome information reported in this paper will provide some useful information for further investigation on the evolution of the family Rosaceae.

## Introduction

**Figure 1.**
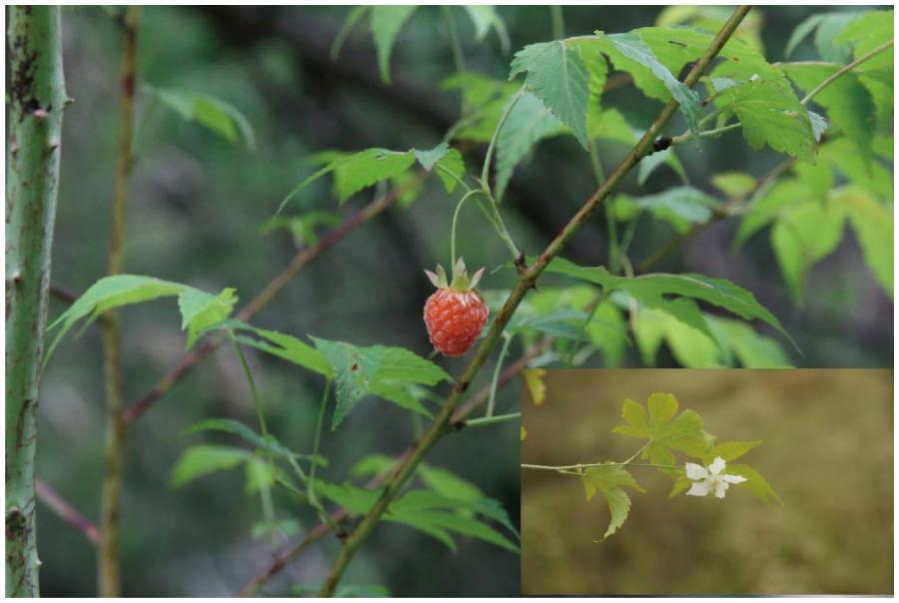
*Rubus chingii* reference image. Photographed by Yongfei Yin. It is a vine like shrub with prickly bark and hairless stems. The leaves are single leaf, nearly circular, palmately 5-lobed, and sparsely 3- or 7-lobed. Stipules persist in a linear lanceolate shape. The flowers are white, and the fruit is red and nearly spherical, with a diameter of 1.5–2 cm. It is densely covered with gray white pubescence.

*Rubus chingii* Hu 1925 is an important perennial medicinal vine shrub of the *Rubus* genus of the Rosaceae family and extensively distributed in Zhejiang, Jiangsu, Anhui, Jiangxi, Fujian, and Guangxi provinces of China and also in Japan (Liu and Niu 2014; Yu et al. 2019) (Figure 1). Its immature fruits named ‘Fu-Pen-zi’ are used as a traditional Chinese medicine with a long history usage listed in Chinese Pharmacopoeia with the function of tonifying the kidney, consolidating semen, and reducing urine. They can be used for kidney deficiency, frequent urination, impotence, premature ejaculation, and spermatorrhea (Xie et al. 2013). Its mature fruits have delicate taste, sweet and sour taste, rich nutrition, and are rich in amino acids, vitamins. In particular, the content of anti-aging substances SOD and trace element Se is higher than that of existing cultivated and wild fruits, which have high nutritional value and medical health care effect (Xie et al. 2013). The World Food and Agriculture Organization (FAO) recommends them as the third generation of fruits in the world. Pharmacological analysis showed that *R. chingii* had many pharmacological effects, such as anti-inflammatory, anti-oxidant, anti-cancer, anti-microbial, and anti-complementary activities (Yu et al. 2019). Terpenoids, flavonoids, steroids, and alkaloids are its main pharmacological active ingredients (He et al. 2022). However, the phylogenetic position of this species requires in-depth analysis due to the complex debate over species identification and classification of the genus *Rubus* (Wang et al. 2021). Here, we characterized the complete chloroplast genome sequence of *R. chingii* according to high throughput sequencing technology. On the one hand, compared with the reported the chloroplast genome of *R. chingii* (Wang et al. 2020), our samples exhibited a certain degree of genetic diversity; on the other hand, more importantly, this study provided more accurate sequence assembly and annotation analysis results, which will provide more accurate references for subsequent research on the phylogenetic and genetic relationships of *R. chingii* and its related species.

## Materials and methods

The *R. chingii* were introduced from Jinzhai county, Lu’an city, Anhui province, China to the Jilin Agricultural Science and Technology University nursery base (43°96′ N, 126°49′ E). We prepared the voucher specimen and deposited it at the Herbarium of Traditional Chinese Medicine Department, Jilin Agricultural Science and Technology University (Yuxian Li, 769831847@qq.com, http://www.zyxy.jlnku.edu.cn, voucher number: JAST2303018). The total genomic DNA of *R. chingii* was extracted from its fresh leaves by the improved CTAB-based method (Li et al. 2013). After testing for DNA concentration and purity, the total DNA was randomly broken, repaired, and connected to construct a sequencing library. The qualified library was used to sequence on the BGISEQ-500 sequencer according to the manufacturer’s instructions detailed in the previous literature (Huang et al. 2017). The clean data totaled 5.33 GB after quality control processing, and yielded a 1335-fold depth of coverage of the chloroplast genome. The quality-controlled clean data were *de novo* assembled into the complete chloroplast genome using NOVOPlasty4.2.1 (Dierckxsens et al. 2017). We used the chloroplast genome of *R. longisepalus* (GenBank ID MW436703) as a reference for assembly. The parameter settings for NOVOPlasty4.2.1 are Genome Range = 150,000–165,000, K-mer = 49, Seed Input and Reference sequence are both MW436703, and other parameters are defaulted. Then, GeSeq (Tillich et al. 2017) and tRNAscan-SE (Lowe and Eddy 1997) were used to annotate the chloroplast genome. Chloroplast genome map was drawn by online software CPGview (Liu et al. 2023). The Mafft software was used to compare genome sequences, and then manually counted SNPs and InDels (Katoh and Standley 2013). The complete chloroplast genome sequence of *R. chingii* was deposited in GenBank with accession number OL449946.

To obtain a clear view of phylogenetic relationship, we downloaded complete chloroplast genomes (full DNA) of 19 other related species from NCBI to reveal the phylogenetic position of *R. chingii* using MAFFT v7.307 (Katoh and Standley 2013) with the default parameters. *Prunus domestica* and *Malus doumeri* were selected as outgroup for the phylogenetic reconstruction. A maximum-likelihood (ML) tree was constructed by FastTree version 2.1.10 (Price et al. 2010) with Generalized Time Reversible (GTR) model and Shimodaira Hasegawa (SH) test.

## Results and discussion

**Figure 2.**
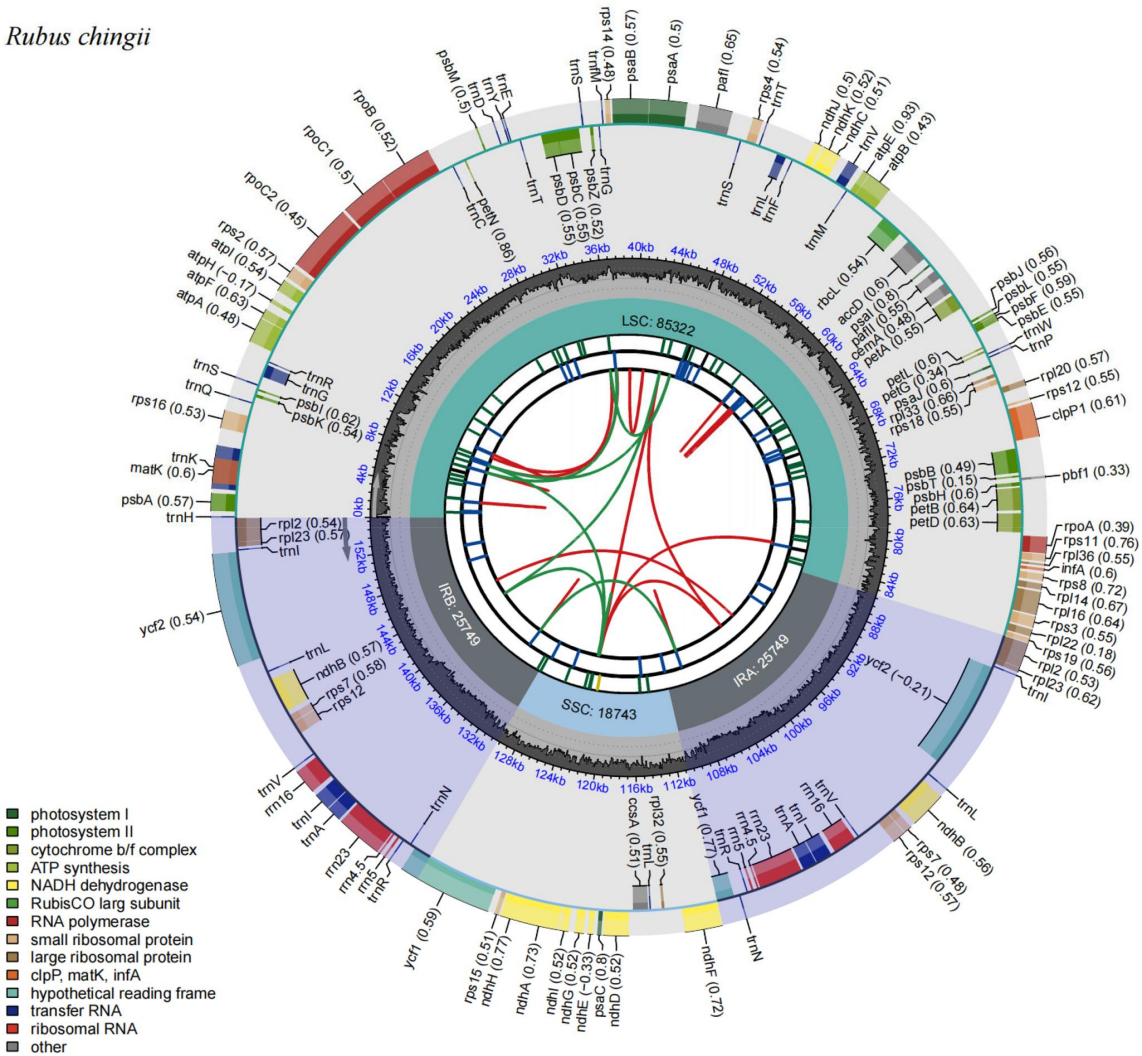
The complete chloroplast genome map of *R. chingii.* It was generated using CPGView. The map has six tracks from the center to the outside. The first track shows the forward and reverse repeats connected with the red and green arcs, respectively. The second track and the third track represent long tandem repeats (blue bands) and short tandem repeats or microsatellites (green bands), respectively. The fourth track shows the GC content and the fifth circle indicates the position of LSC, SSC, IRA, and IRB. The sixth track represents the genes as colored boxes, the inner boxes mean clockwise transcription, and the outer boxes mean counterclockwise transcribed genes.

The chloroplast genome of *R. chingii* was 155,563 bp in length, consisting of two single-copy regions (a large single-copy (LSC) and a small single-copy (SSC) regions) of 85,322 bp and 18,743 bp, respectively, which were separated by a pair of 25,749 bp inverted repeat (IR) regions (Figure 2). The GC content of the genome was 37.06%. It was 34.94%, 30.83%, and 42.84% in LSC, SSC, and IR regions, respectively. A total of 131 genes were annotated, including 86 protein-coding genes, 37 tRNA genes, and eight rRNA genes. Within the *R. chingii* chloroplast genome, six protein-coding genes, eight tRNA genes, and four rRNA genes were duplicated in IR regions. Eighteen genes had two exons and four genes (*clp*P1, *paf*I, and two *rps*12) contained three exons. The *rps*16, *rpo*C1, *paf*I, *clp*P1, *pet*B, *pet*D, *rpl*16, *rpl*2, *ndh*B, and *ndh*A genes were cis-splicing genes (Figures S1 and S2). In addition, one of the most important findings is that through this study, there were some differences in annotation and assembly of the chloroplast genome of *R. chingii* in previous reports (Wang et al. 2020). Specifically, one major difference might be existed in the assembly of *rpl*2, which is located in the IR region. Normally, this sequence is exactly the same in the IRA and IRB regions. However, the publicly available *rpl*2 sequence assembles an additional segment in the IRA region, and the entry and exit segments also contain *rps*19 (Figure S3). The results of our assembly are completely consistent in the IRA and IRB regions, consistent with the normal characteristics of this genus and plant chloroplasts. Moreover, the read coverage map also showed that the read coverage was relatively uniform (Figure S4), indicating that the data quality is reliable in this study. It is noteworthy that 338 SNPs and InDels were identified by comparing with the chloroplast genome of *R. chingii* previously reported (GenBank ID MN885523), and the longest InDel was 64 bp, which indicated that *R. chingii* displayed a high genetic diversity.

**Figure 3.**
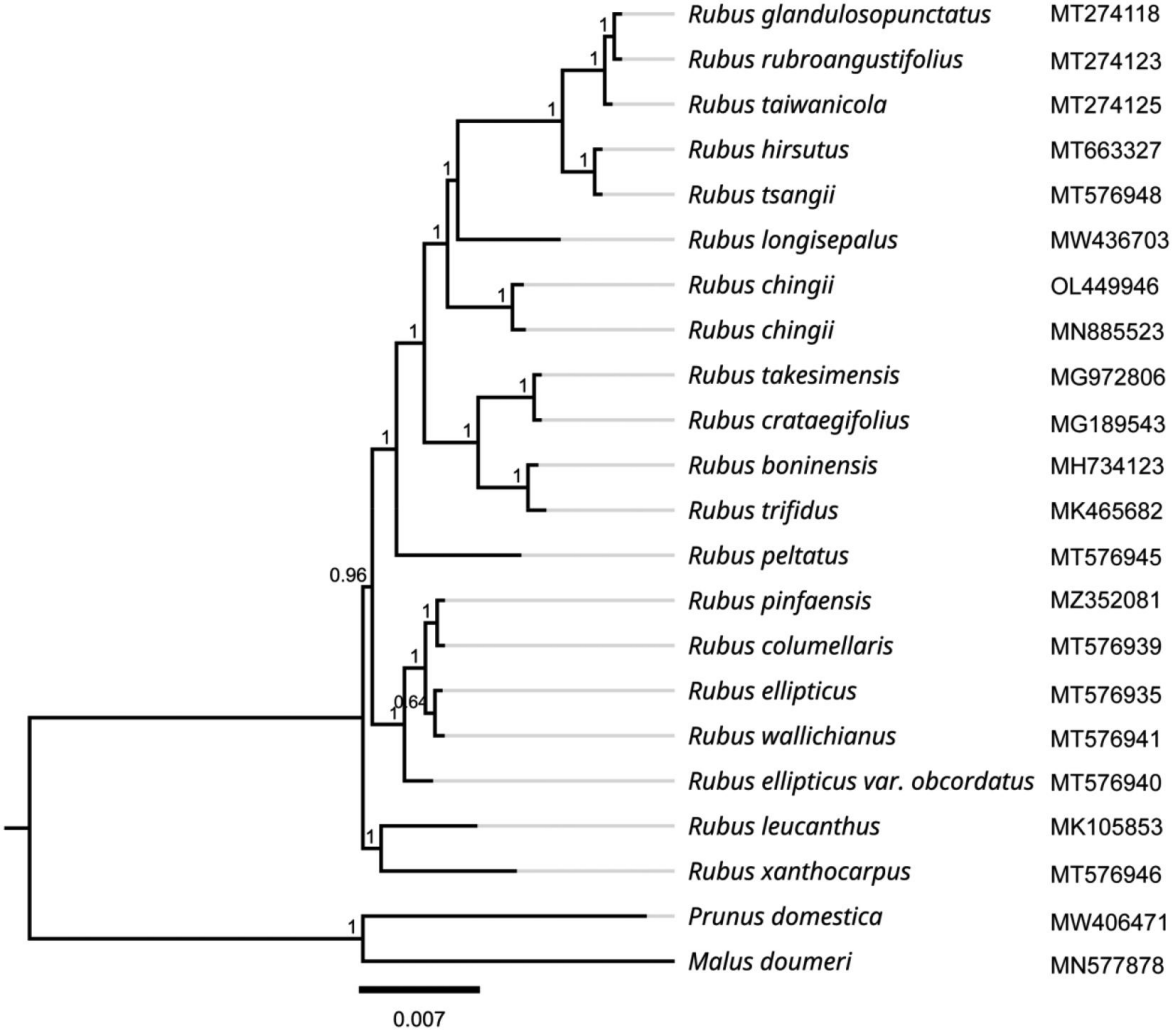
Phylogenetic tree inferred by maximum-likelihood (ML) method based on 20 representative species. *Prunus domestica* and *Malus doumeri* were used as outgroup taxa. A total of 1000 bootstrap replicates were computed and the bootstrap support values are shown at the branches. The gray lines were used to supplement the branch length, and the branch length of the phylogram was black. The following sequences were used: *Rubus glandulosopunctatus* MT274118 (Yang et al. 2021), *Rubus rubroangustifolius* MT274123 (Yang et al. 2021), *Rubus taiwanicola* MT274125 (Yang et al. 2021), *Rubus hirsutus* MT663327 (Wang et al. 2021), *Rubus tsangii* MT576948, *Rubus longisepalus* MW436703 (Park et al. 2021), *Rubus chingii* OL449946, *Rubus chingii* MN885523 (Wang et al. 2020), *Rubus takesimensis* MG972806 (Yang et al. 2018), *Rubus crataegifolius* MG189543 (Yang et al. 2017), *Rubus boninensis* MH734123 (Yang et al. 2017), *Rubus trifidus* MK465682 (Yang et al. 2017), *Rubus peltatus* MT576945 (Yang et al. 2018), *Rubus pinfaensis* MZ352081, *Rubus columellaris* MT576939, *Rubus ellipticus* MT576935 (Zhu et al. 2022), *Rubus wallichianus* MT576941, *Rubus ellipticus* var. *Obcordatus* MT576940, *Rubus leucanthus* MK105853 (Guo et al. 2019), *Rubus xanthocarpus* MT576946, *Prunus domestica* MW406471 (Geng et al. 2020), and *Malus doumeri* MN577878 (Wang et al. 2019).

To explore the phylogenetic relationship among *R. chingii* and its related taxa, the complete chloroplast genomes from 19 other related species downloaded from the NCBI GenBank database. As shown in Figure 3, the ML analysis produced a phylogenetic tree which displayed *Rubus chingii* and several other *R. longisepalus*, *R. tsangii*, *R. hirsutus*, *R. taiwanicola*, *R. rubroangustifolius*, and *R. glandulosopunctatus* together formed the monophylic group, indicating that they might have a common ancestor. The genomic information reported in this study will be beneficial for further enriching in-depth and precise research on the evolution of this species.

## Conclusions

In this study, we sequenced and identified the complete chloroplast genome of *R. chingii*. The analysis results of phylogenetic relationship showed that *R. chingii* formed the monophylic group with *R. longisepalus*, *R. tsangii*, *R. hirsutus*, *R. taiwanicola*, *R. rubroangustifolius*, and *R. glandulosopunctatus* of genus *Rubus*. This study laid the foundation for providing valuable information that can contribute to the identification and further evolutionary analysis and genetic improvement of this species.

## Supplementary Material

Supplemental Material

Supplemental Material

Supplemental Material

Supplemental Material

## Data Availability

The genome sequence data of *R. chingii* that support the findings of this study are openly available in GenBank of NCBI under the accession no. OL449946. The associated BioProject, SRA, and Bio-Sample numbers are PRJNA782849, SRR17013867, and SAMN23401999, respectively.
