# Supplementary material for: Characteristics and phylogenetic analysis of the complete chloroplast genome of Rubus chingii Hu 1925 from the family Rosaceae
Source: Mitochondrial DNA B Resour. 2023 Nov 20;8(11):1280–4. doi: 10.1080/23802359.2023.2268220 (PMC10986437; doi:10.1080/23802359.2023.2268220)
Supplement: Supplemental Material [file TMDN_A_2268220_SM4974.docx]

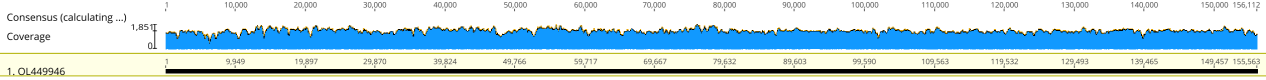


Figure S4. Overall coverage depth of the chloroplast genome assembly of *Rubus chingii*. This figure was generated using Geneious Prime by aligning DNA-Seq data to the whole chloroplast genome. The height of the blue graph indicates the number of sequences at each location.
