# Supplementary material for: Characteristics and phylogenetic analysis of the complete chloroplast genome of Rubus chingii Hu 1925 from the family Rosaceae
Source: Mitochondrial DNA B Resour. 2023 Nov 20;8(11):1280–4. doi: 10.1080/23802359.2023.2268220 (PMC10986437; doi:10.1080/23802359.2023.2268220)
Supplement: Supplemental Material [file TMDN_A_2268220_SM4971.docx]

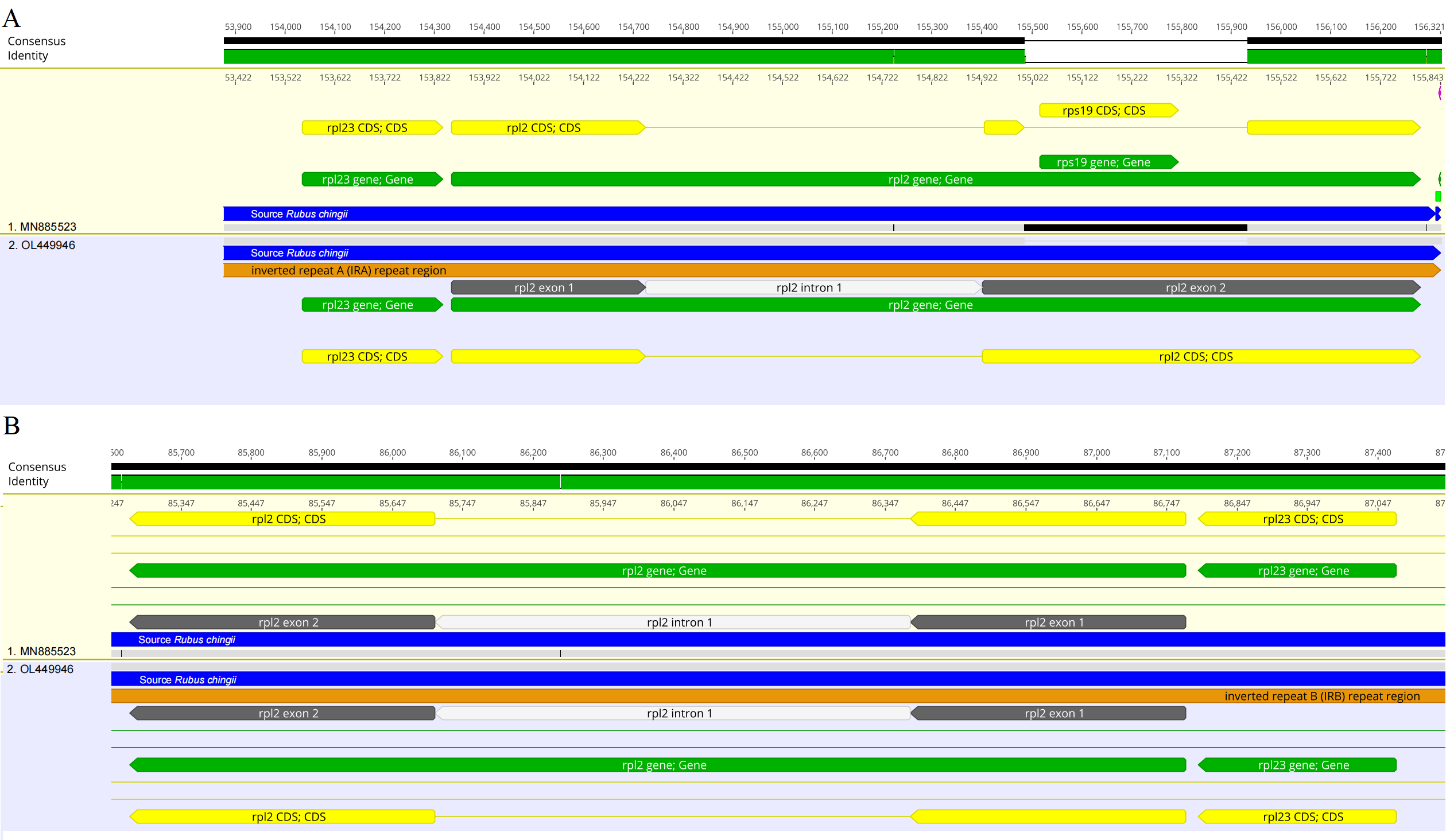


Figure S3. The distribution of *rpl*2 gene in IRA (A) and IRB (B) regions of the two chloroplast genome of *R. chingii.* 1 represents previously reported (GenBank number MN885523), and 2 represents in this study (GenBank number OL449946).
