# Supplementary material for: Characteristics and phylogenetic analysis of the complete chloroplast genome of Rubus chingii Hu 1925 from the family Rosaceae
Source: Mitochondrial DNA B Resour. 2023 Nov 20;8(11):1280–4. doi: 10.1080/23802359.2023.2268220 (PMC10986437; doi:10.1080/23802359.2023.2268220)
Supplement: Supplemental Material [file TMDN_A_2268220_SM4936.docx]

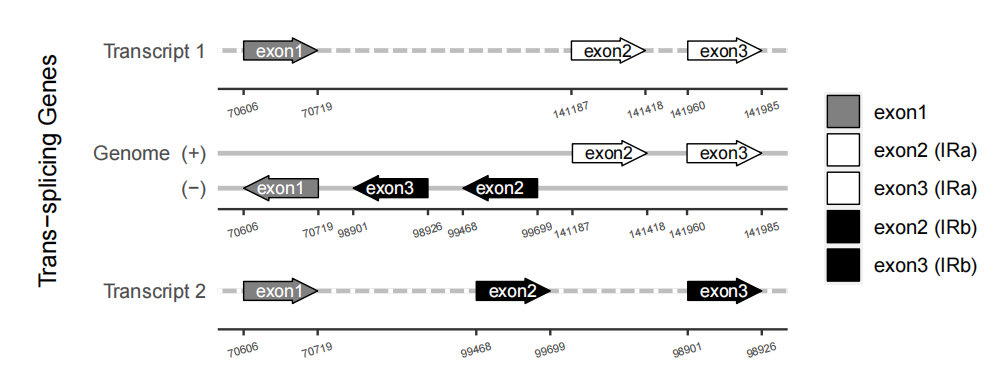


Figure S2. Schematic map of trans-splicing gene *rps*12 in the chloroplast genome of *Rubus chingii*. The arrow indicates the sense direction of gene. The map was generated using CPGview.
