# Supplementary material for: Characteristics and phylogenetic analysis of the complete chloroplast genome of Rubus chingii Hu 1925 from the family Rosaceae
Source: Mitochondrial DNA B Resour. 2023 Nov 20;8(11):1280–4. doi: 10.1080/23802359.2023.2268220 (PMC10986437; doi:10.1080/23802359.2023.2268220)
Supplement: Supplemental Material [file TMDN_A_2268220_SM4935.docx]

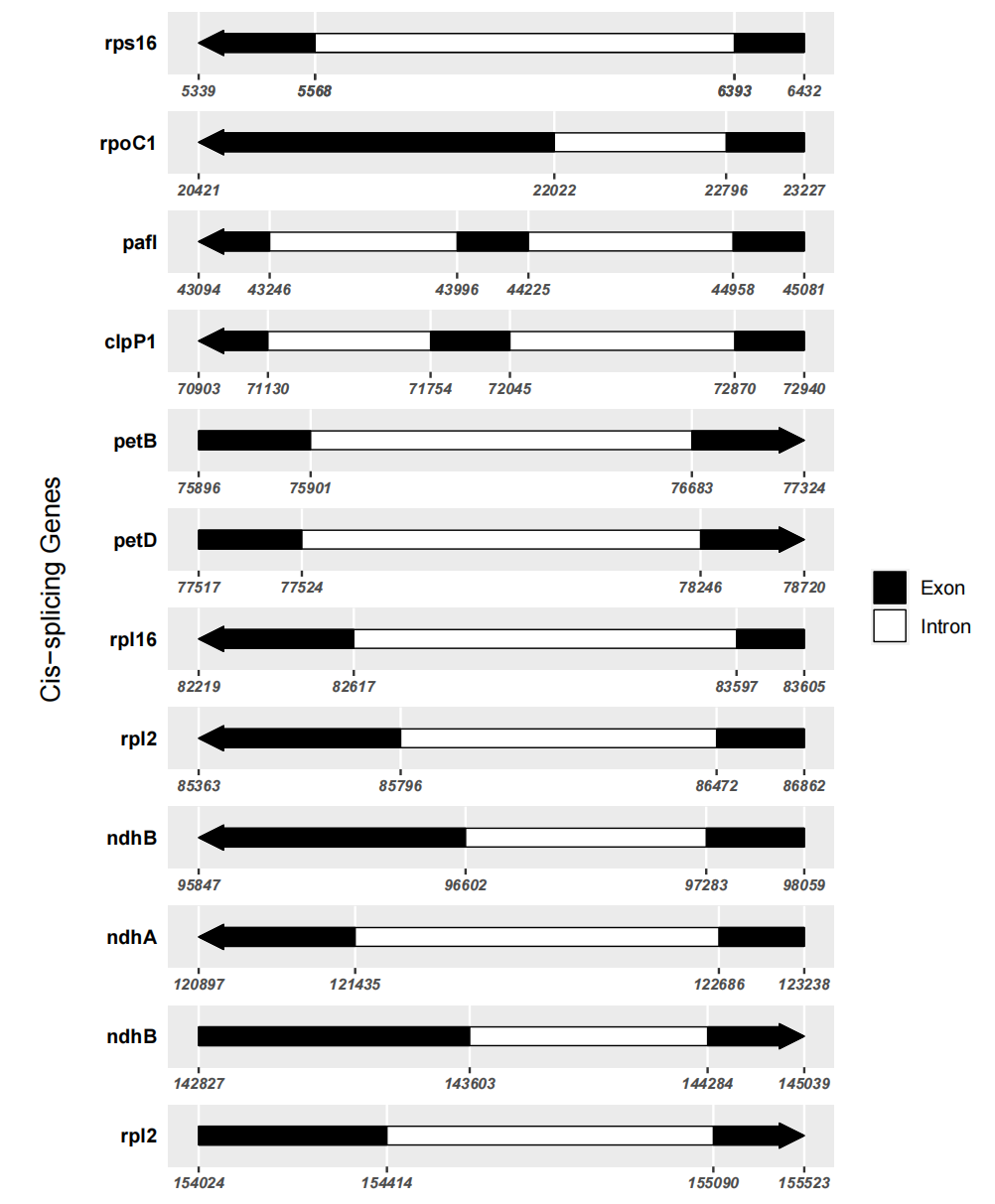


Figure S1. Schematic map of the cis-splicing genes in the chloroplast genome of *Rubus chingii*. Black is exon and white is intron. The map was generated using CPGview.
